# Wide-field swept-source OCTA in the assessment of retinal microvasculature in early-stage diabetic retinopathy

**DOI:** 10.1186/s12886-022-02724-0

**Published:** 2022-12-06

**Authors:** Xiang-ning Wang, Xuan Cai, Shi-wei Li, Tingting Li, Da Long, Qiang Wu

**Affiliations:** 1 Department of Ophthalmology, Shanghai Sixth People’s Hospital, 600 Yishan Road, Shanghai, 200233 Xuhui District China; 2 Shanghai Key Laboratory of Diabetes Mellitus, Shanghai, 200233 China

**Keywords:** Diabetic retinopathy, OCTA, Perfusion area, Swept-source, Vessel density

## Abstract

**Background:**

To perform a quantitative analysis of retinal microvasculature in patients with early-stage diabetic retinopathy (DR) using wide-field swept-source optical coherence tomography angiography (SS-OCTA).

**Methods:**

One hundred nineteen eyes of 119 patents (67 eyes with no DR and 52 eyes with mild-moderate nonproliferative diabetic retinopathy (NPDR)) were enrolled in this observational and cross-sectional cohort study, and an age-matched group consisting of 39 eyes of 39 non-diabetic subjects were set as the control. Each participant underwent a full ophthalmic examination, including wide-field SS-OCTA imaging. On OCTA scans (12 mm * 12 mm), the mean perfusion area (PA) and vessel density (VD) were independently measured in all 16 Early Treatment Diabetic Retinopathy Study (ETDRS) sectors. Linear regression analyses were conducted to evaluate the influences of PA.

**Results:**

In the central ring, there were no significant differences in the average PA and VD among the groups. In the 3 mm radius, the PA and VD of the no DR and mild-moderate NPDR were significantly decreased compared with the control group in superior and inferior quadrants. In the wide-field scans (9 and 12 mm radius), there was no significant difference in average PA and VD between the groups in each sectors (*p* > 0.05). Regression analysis found that the effect of VD on PA was statistically different (b = 1.311, *p* < 0.001).

**Conclusion:**

Wide-field OCTA imaging is useful for evaluating peripheral capillary perfusion in eyes with early-stage DR. Decrease in PA and VD was greater in the S3 and I3 sectors, and reductions in PA and VD were uneven in wide-filed sectors (9 and 12 mm radius).

## Background

Diabetic retinopathy (DR) is one of the most frequent microvascular complications of diabetes [1]. Approximately 34.6% of people are living with DR, and 7% have vision-threatening DR. Also, the global population affected by diabetes is expected to reach 366 million people by 2030 [2, 3]. According to pathophysiological research, neuronal disease and neurodegeneration are highly associated with microvascular dysfunction, and neurovascular unit degeneration can be considered a significant component of the pathology of DR [4, 5]. Furthermore, changes in the retinal microcirculation appear in diabetic eyes before clinically evident retinopathy develops [6, 7]. Moreover, harm to the retinal capillaries caused by retinal ischemia is manifested clinically as retinal capillary nonperfusion and expansion of the foveal avascular zone (FAZ) [8]. Quantifying retinal ischemia in the early stage of DR can provide the clinicians with key information for determining the severity and progression of the disease [9].

Color fundus photography (CFP) and fluorescein angiography (FA) are commonly used to assess and schedule care for various phases of DR. FA is the current gold standard for evaluating retinal capillary occlusion in various retinal vascular diseases [10]. However, FA is an invasive and time-consuming procedure associated with certain adverse reactions. Furthermore, FA is very useful for observing larger vessels as well as alterations in vascular permeability, but is not well suited for detecting early changes in the microvasculature [11].

The non-invasive technology of optical coherence tomography angiography (OCTA) has revolutionized the imaging of the retinal and choroidal microvasculature [12]. Its high-resolution microvascular detail enables simple detection of the FAZ in eyes with DR. Importantly, capillary changes can be observed even in the early stages [13]. However, most modern OCTA machines can only imagine a narrow field of view around the macula (3*3 mm or 6*6 mm) and provide little insight into the peripheral regions. Since vascular changes in the peripheral region of the eye are more common in DR, looking into a narrow field of view can lead to misclassification [14]. Several machines have recently implemented wide field of view protocols, and by stitching or inserting an ocular lens, one can achieve a wider field of view up to a 20*20-mm2 field [15–17]. Yet, these methods have not been validated in larger populations.

OCTA depiction of retinal vasculature over 12*12 mm and 15*9 mm fields of view is now possible thanks to the latest invention of commercially available swept-source OCT (SS-OCT) instruments with 100-kHz A-scan frequencies, partly bridging the difference between OCTA and FA field sizes [15, 18, 19]. The aim of this study was to quantitatively analyze the retinal microvasculature using wide-field SS-OCTA in patients with early-stage DR.

## Methods

### Participants

The study protocol was approved by the Institutional Review Board of Shanghai Sixth People’s Hospital. All procedures preceded the Declaration of Helsinki's tenets, and all participants gave their informed consent before participating in the study. It was also registered in the Chinese clinical trial registry (http://www.chictr.org.cn/, Registration number: ChiCTR1900028607). Between October 2021 and March 2022, patients with type 2 diabetes mellitus (T2DM) were recruited from Shanghai Diabetes Centre at Shanghai Jiaotong University Affiliated Sixth People's Hospital. During the same time, age-matched healthy controls were recruited from the hospital's medical assessment center.

Each of the participants completed a comprehensive ophthalmologic test, which included taking a medical history, determining visual acuity, biomicroscopy of the anterior segment with a slit light, ophthalmoscopy of the posterior segment, and wide-field colour fundus imaging with the Optos camera (Optos plc, Dunfermline, United Kingdom) and wide-field SS-OCTA instrument (VG200S; SVision Imaging, Ltd., Henan, China). Two senior researchers (XC and TTL) used the International Clinical Diabetic Retinopathy Disease Severity Scale to classify subjects using wide-field color fundus photography [20]; as no DR, mild-moderate NPDR, severe non-proliferative DR (NPDR), or proliferative DR (PDR). Each subject had one eye chosen as the research eye, and if both eyes matched the eligibility criterion, the eye with higher visual acuity or a lower refractive error was chosen. All of the participants were ≥ 18 years old, had a log MAR visual acuity ≤ 0.5 log units, had a refractive error of less than -3.00 diopters or + 3 diopter-equivalent spheres, and had a DR classification of less than severe NPDR. The exclusion criteria were: (1) unable to give informed consent or perform a full examination; (2) pregnant or nursing; (3) the history of any ocular diseases (except for age-related cataract), hypertensive retinopathy, and history of any ocular surgeries (except for cataract surgery); (4) the presence of diabetic macular edema (DME).

### SS-OCT/OCTA image acquisition and analysis

An SS laser with a central wavelength of approximately 1050 nm and a scan rate of 200,000 A-scans per second was used in the SS-OCT/OCTA method (VG200S; SVision Imaging, Henan, China). The device had an eye-tracking utility built on an optimized confocal scanning laser ophthalmoscope to remove eye-motion objects. The axial and lateral resolutions were 5 μm and 13 μm, respectively. The scan depth was 3 mm.

The fovea was based on OCTA volumes occupying a 12*12 mm retinal region (40° field of view). Each 12*12 mm volume had 1024 A-scans per B-scan (24 mm spacing between adjacent A-scans) and 1024 B-scan positions per volume scan (24 mm spacing between adjacent B-scans); OCTA images were produced by acquiring two replicated B-scans at each B-scan spot. At least 12 s were necessary to obtain a single 12*12 mm volume. Professional ophthalmic photographers performed all OCTA scanning, and where necessary, image acquisitions were replicated many times to ensure photographs of high OCT signal penetration and minimum motion artifacts. The scans’ quality scores were expressed as an SNR in decibels (dB) on a scale of 1 (poor quality) to 10 (excellent quality), with scans scoring > 8 dB considered good quality and included.

### Image analysis

**Fig. 1:**
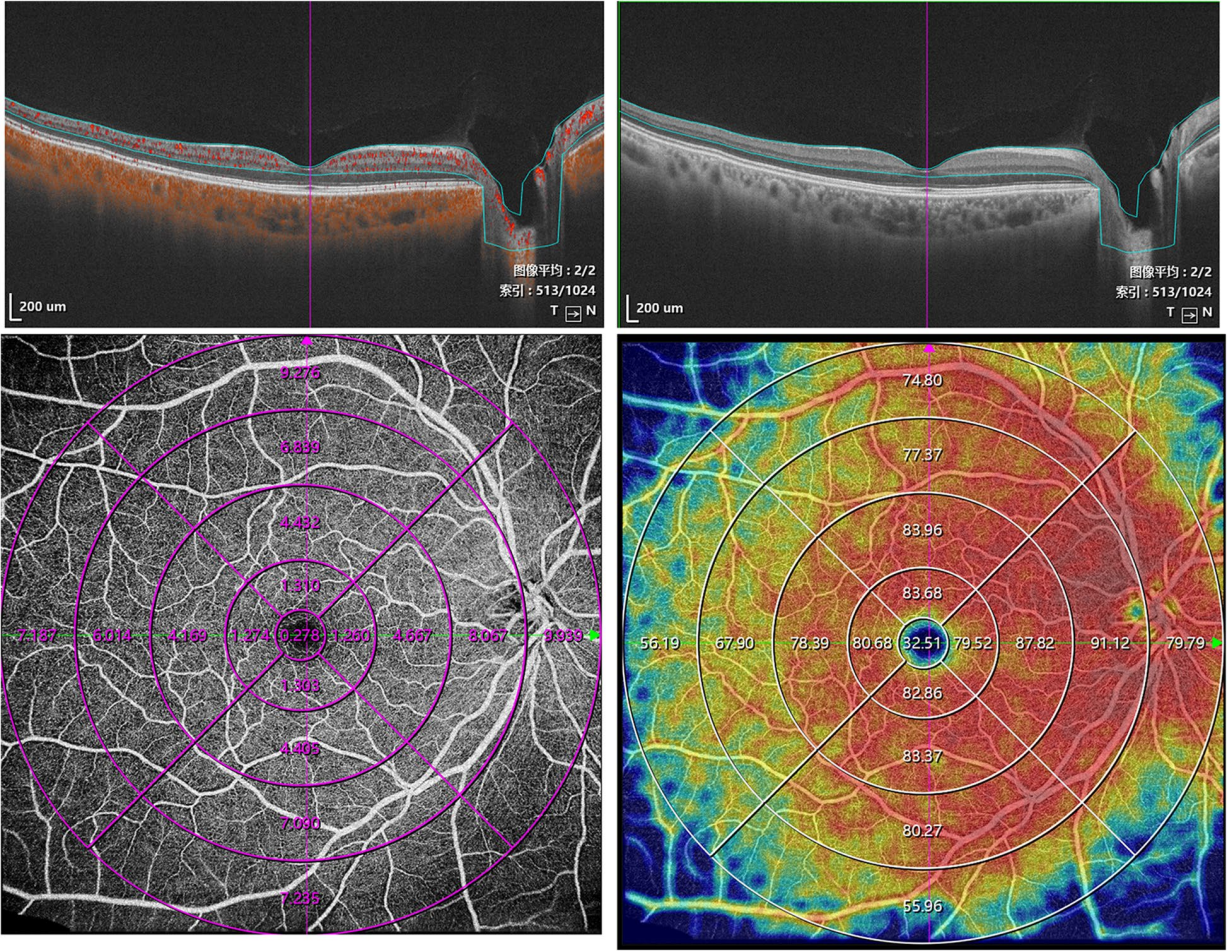
Representative SS-OCT/OCTA scans with perfusion area and vascular density map (ETDRS grid)

First, the location of the macular fovea was determined by adjusting the horizontal and vertical markings of the central area of the macula. The two markings were located in the central fovea (lowest area) of the B-SCAN (Fig. 1). The intersection of the two markings could determine the position of the central fovea. Second, the Early Treatment Diabetic Retinopathy Study (ETDRS) subfield measurements were performed through the built-in mapping software. The topographic map's center was rotated to coincide with the center of the above axis. Next, the data were automatically averaged through the following subfields and sectors: the central fovea subfield within the inner 1-mm-diameter circle; the circle subfield between (1–3 mm)-diameter circles, (3–6 mm)-diameter circles, (6–9 mm)-diameter circles and (9–12 mm)-diameter circles. Also, each circles was sectioned into superior, nasal, inferior, and temporal quadrants. Finally, each patterns of average perfusion area (PA) and blood vessel density (VD) of the inner retina (from ILM-5 to IPL/INL + (INL/OPL—IPL/INL)/2) were calculated using the built-in software software (version 1.31.6; VG200D, SVision Imaging, Ltd.) (Fig. 1).

### Statistical analysis

SPSS program, version 27.0, was used for conduct all of the data analyses (IBM Corporation, Chicago, IL, USA). The quantitative variables were expressed as the means, standard deviation (SD), and maximal and minimal values. The qualitative variables were expressed in absolute and relative frequencies. Normality was assessed using the Shapiro–Wilk test, and the non-Gaussian distribution was determined. The nonparametric Kruskal–Wallis with Bonferroni’s post hoc test was used to compare the numerical variables obtained between the study group and the control groups. A comparison of different areas in the same ring was made using the paired test. Spearman’s correlation coefficient was used to evaluate the correlation between the average PA and variables, including age, refractive error, duration of diabetes, fasting blood glucose (FBG), HbA1c and GA. All of the recorded p-values were two-tailed. To determine statistical significance, a form I error level of 0.05 was used. A *p*-value < 0.05 was considered statistically significant.

## Results

After screening according to the inclusion/exclusion criteria, 158 participants were included in the research (39 healthy controls and 119 diabetes patients; 79 men and 79 women), 39 of whom were classified as controls, 62 as having T2DM without DR (no-DR group), and 52 as having mild to moderate NPDR (mild-moderate NPDR). The demographic characteristics of the study participants are shown in Table 1.

**Table 1:** Demographic and clinical characteristics of the study participants

Characteristics	control	no- DR	*P*1 value	mild DR	*P*2 value
Number	39	67	-	52	-
Age, y	60.89 ± 9.87	61.09 ± 10.69	0.928	60.00 ± 10.46	0.668
Gender(male/female)	15/24	38/29	0.07	25/27	0.360
Laterality(right/left)	23/16	39/28	0.939	29/23	0.76
Visual acuity, log MAR	0.12 ± 0.28	0.10 ± 0.18	0.651	0.14 ± 0.14	0.311
Refractive error, diopters	0.56 ± 1.42	-0.08 ± 1.38	0.551	-0.37 ± 1.75	0.629
Duration of diabetes, y	-	9.43 ± 6.48	-	17.14 ± 6.85	< 0.05
FBG,mmoL/L	-	6.74 ± 1.32	-	7.20 ± 1.55	0.292
HbA1c, %		7.30 ± 0.96	-	7.41 ± 0.73	0.766
Glycated Albumin,%		17.79 ± 5.65	-	19.10 ± 7.39	0.435

There was no difference in age, gender, laterality, visual acuity, and refractive error among the groups (all *p* > 0.05). Among the diabetic patients, a comparison of the differences (duration of diabetes, FBG, HbA1c and GA) between the mild-moderate NPDR and no-DR groups showed that only the duration of diabetes was significantly different (T = -5.39 and *p* < 0.05).

### Repeatability

The repeatability test was performed on 12 patients who were 60.12 (6.68) years old on average (range, 50–74 years). Six of the people who took part in the test had diabetes. PA received an ICC score of 0.91, while VD received a score of 0.90.

### Perfusion area (PA) and vascular density measurements

As the distance from the central ring to the outside increased, the PA of the retina also increased. In addition, the PA of the retina increased as the distance between the central ring and the outside increased. Except for the 12 mm scale, where the superior quadrant was greater than the inferior quadrant (*p* < 0.001, Table 2), there was no gap in the PA of the superior and inferior ETDRS regions at the same distance in the control group. In the horizontal direction, the PA of the nasal quadrant at the same distance was larger than that of the temporal quadrant, except in the range of 3 mm (*p* = 0.051, Table 2). The same finding was also observed in the VD.

**Table 2:** Perfusion area and vascular density in ETDRS grid for control group and diabetic group

			Superior	Inferior	P	Temporal	Nasal	*P*
Perfusion area(mm^2^)	Control group	3 mm radius	0.79(0.68,0.85)	0.75(0.63,0.85)	0.172	0.76(0.61,0.84)	0.73(0.63,0.79)	0.051
		6 mm radius	2.89(2.46,3.19)	2.82(2.45,3.17)	0.850	2.80(2.34,3.10)	2.90(2.59.3.25)	0.014
		9 mm radius	4.64(3.99,5.15)	4.38(3.76,5.08)	0.307	4.21(3.52,4.66)	4.94(4.54.5.37)	< 0.01
		12 mm radius	5.89(4.99,6.48)	4.77(4.03,5.61)	< 0.01	4.92(3.82,5.53)	5.75(4.95,6.16)	0.004
	Diabetic patients	3 mm radius	0.66(0.54,0.79)	0.65(0.54,0.78)	0.021	0.67(0.53,0.77)	0.64(0.51,0.73)	0.001
		6 mm radius	2.60(2.17,2.98)	2.64(2.21,2.98)	0.731	2.63(2.17,3.00)	2.75(2.42,3.21)	< 0.001
		9 mm radius	4.07(3.48,4.47)	4.48(3.94,4.93)	0.414	4.07(3.48,4.47)	5.02(4.37,5.70)	< 0.001
		12 mm radius	5,64(4.85,6.27)	4.80(4.02,5.33)	< 0.001	4.60(3.69,5.39)	5.64(4.88,6.46)	< 0.001
Vascular density(%)	Control group	3 mm radius	70.47(64.89,74.86)	69.73(62.89,72.67)	0.232	66.86(62.54,72.67)	66.68(62.43,70.51)	0.113
		6 mm radius	73.69(68.06,76.92)	70.97(68.74,77.22)	0.405	70.65(66.80,74.72)	74.97(68.74,77.22)	< 0.01
		9 mm radius	70.44(64.43,73.14)	68.40(62.12,75.07)	0.330	65.79(57.17,68.76)	76.55(70.15,80.59)	< 0.01
		12 mm radius	61.65(56.92,66.54)	51.96(45.93,59.77)	< 0.01	51.27(39.65,59.48)	62.33(57.19,67.22)	< 0.01
	Diabetic patients	3 mm radius	63.20(58.27,70.67)	62.82(54.76,69.05)	0.011	62.23(54.10,68.56)	61.62(53.57,67.39)	0.167
		6 mm radius	68.77(61.35,74.87)	68.27(60.26,75.17)	0.743	67.33(60.81,73.05)	72.92(68.15,79.74)	< 0.001
		9 mm radius	67.41(61.93,72.93)	66.91(61.44,72.39)	0.960	61.32(55.97,66.70)	76.46(69.71,82.46)	< 0.001
		12 mm radius	61.15(55.13,67.13)	52.00(46.50,58.01)	< 0.001	48.57(40.43,55.97)	63.07(55.02,68.79)	< 0.001

Among the diabetic patients, except in the range of 6 mm and 9 mm, where the discrepancy between the two was not statistically important (*p* = 0.731 and 0.414, Table 2), the PA of the superior quadrant at the same distance was greater than that of the inferior quadrant for diabetic patients. In the horizontal direction, the PA of the nasal quadrant at the same distance was larger than that of the temporal quadrant, the difference between the two was statistically significant (*p* < 0.05, Table 2). The same result was seen for VD, except that the difference between the two was not statistically significant (*p* = 0.167, Table 2) in the 3 T and 3 N regions.

**Fig. 2:**
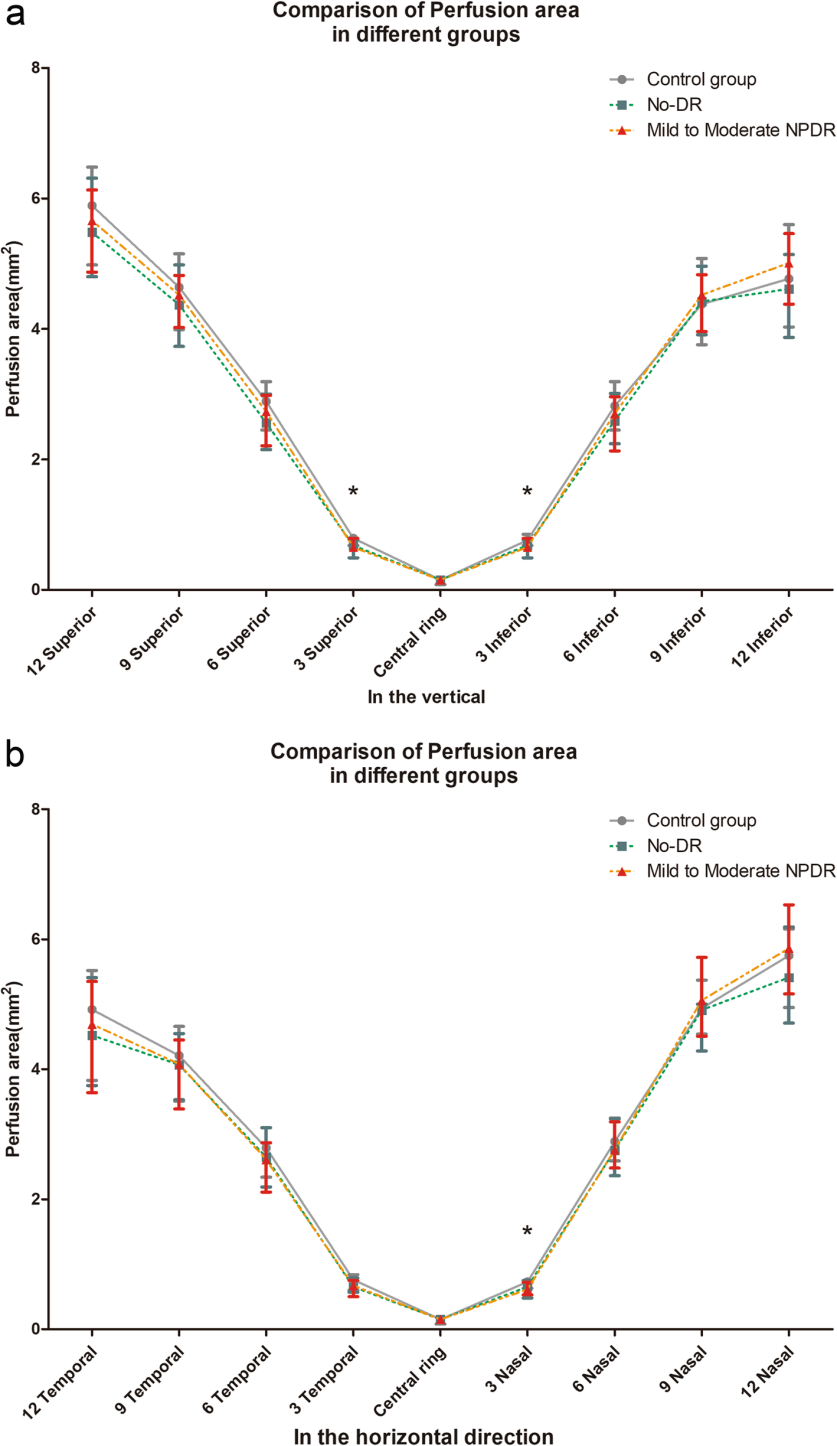
Comparison of perfusion area in different groups in the vertical direction (**a**) and in the horizontal direction (**b**)

The average PA was compared among the three groups. Sectored analyses of each layer's thickness in the ETDRS grid are shown in Tables 3 and Fig. 2. In the central ring, the PA of the control group, no-DR and mild-moderate NPDR groups were 0.15 (0.08, 0.19), 0.15 (0.11, 0.20), and 0.15 (0.10, 0.19)mm^2^, respectively, without significant difference (*p* = 0.953). In the 3 mm radius, there were differences in the superior, inferior and nasal quadrants (*p* = 0.023, 0.029, 0.026). Among them, the PA of the control group was bigger than the no-DR and mild-moderate NPDR groups (*p* < 0.05). However, the difference between the no-DR and mild-moderate NPDR groups was not statistically significant (*p* = 0.606, 0.867, 0.9111). In the wide-field scans (6 mm, 9 mm, and 12 mm radius), the average PA showed no significant difference between the groups in the ETDRS grid (*p* > 0.05).

**Table 3:** Comparison of perfusion area parameters between each group

	Control group	No-DR	Mild-Moderate NPDR	*P*	1 vs 2	1 vs 3	2 vs 3
Central ring	0.15(0.08,0.19)	0.15(0.11,0.20)	0.15(0.10,0.19)	0.953	0.864	0.974	1
3 mm radius							
Superior	0.79(0.68,0.85)	0.68(0.49,0.77)	0.65(0.61,0.79)	0.023	0.024	0.013	0.606
Inferior	0.76(0.63,0.85)	0.66(0.49,0.79)	0.66(0.49,0.79)	0.029	0.049	0.046	0.867
Temporal	0.76(0.60,0.84)	0.65(0.57,0.79)	0.67(0.50,0.75)	0.099	0.166	0.260	0.408
Nasal	0.73(0.63,0.78)	0.65(0.48,0.75)	0.61(0.53,0.72)	0.026	0.047	0.045	0.911
6 mm radius							
Superior	2.89(2.45,3.19)	2.56(2.15,3.00)	2.73(2.21,2.98)	0.108	0.420	0.098	0.709
Inferior	2.82(2.45,3.01)	2.59(2.24,3.01)	2.70(2.13,2.96)	0.153	0.093	0.073	0.876
Temporal	2.79(2.34,3.10)	2.65(2.19,3.10)	2.61(2.11,2.87)	0.221	0.585	0.086	0.220
Nasal	2.89(2.59,3.25)	2.75(2.36,3.24)	2.76(2.48,3.19)	0.595	0.327	0.511	0.660
9 mm radius							
Superior	4.64(3.99,5.15)	4.37(3.73,4.98)	4.52(4.02,4.82)	0.393	0.205	0.416	0.455
Inferior	4.38(3.76.5.08)	4.42(3.91,4.96)	4.52(3.96,4.83)	0.974	0.856	0.813	0.969
Temporal	4.21(3.51,4.66)	4.07(3.53,4.55)	4.08(3.39,4.45)	0.445	0.549	0.195	0.455
Nasal	4.94(4.54,5.37)	4.91(4.28,5.60)	5.06(4.51,5.72)	0.603	0.742	0.381	0.416
12 mm radius							
Superior	5.89(4.98,6.48)	5.48(4.80,6.31)	5.66(4.87,6.13)	0.667	0.429	0.411	0.902
Inferior	4.77(4.03,5.60)	4.61(3.87,5.14)	5.01(4.38,5.46)	0.086	0.220	0.552	0.028
Temporal	4.92(3.83,5.52)	4.52(3.75,5.41)	4.69(3.64,5.35)	0.828	0.585	0.581	0.924
Nasal	5.75(4.95,6.16)	5.41(4.71,6.19)	5.86(5.16,6.53)	0.068	0.425	0.107	0.032
0-12 mm radius	53.86(46.69,58.81)	51.90(46.87,55.99)	51.86(48.83,56.57)	0.422	0.358	0.487	0.507

**Fig. 3:**
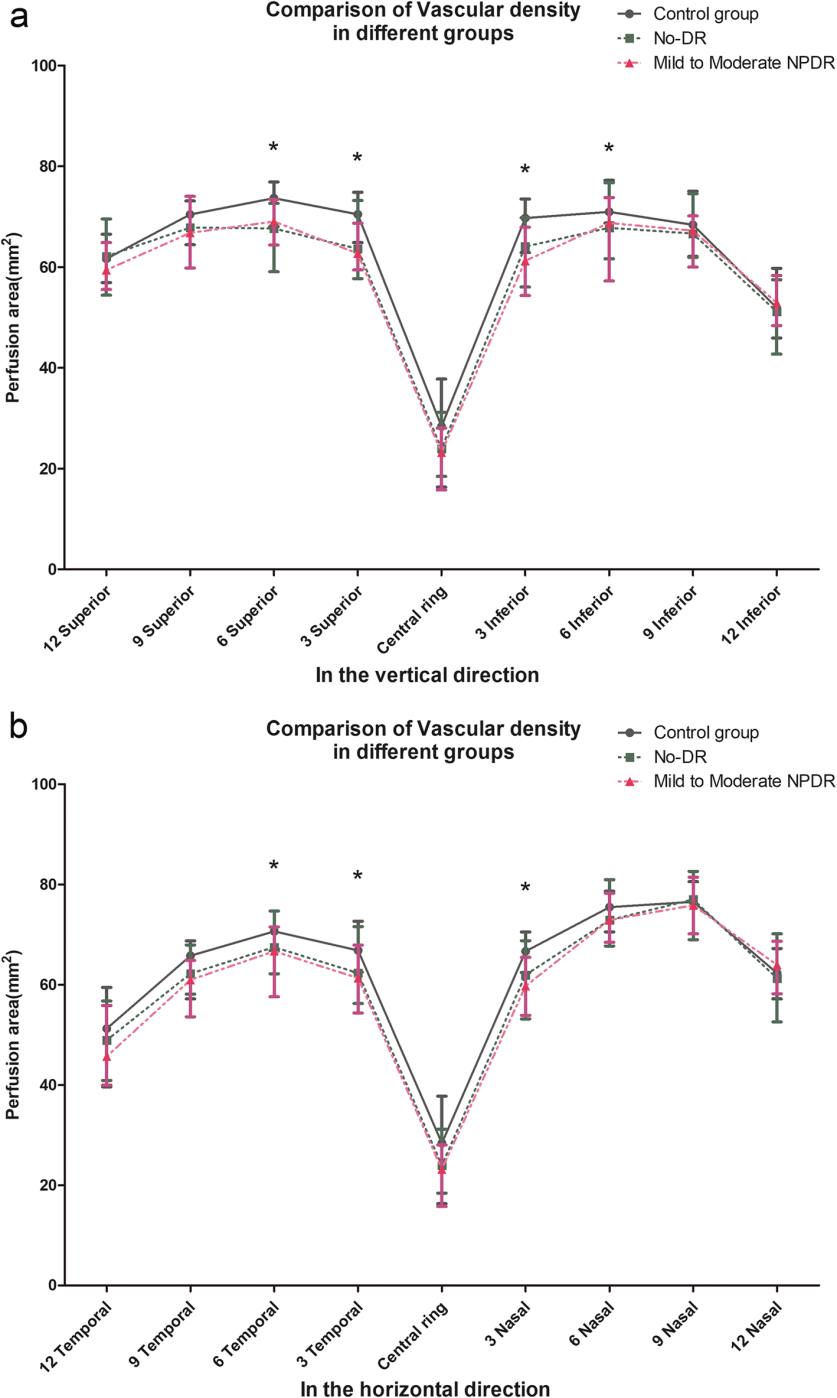
Comparison of vascular density in different groups in the vertical direction (**a**) and in the horizontal direction (**b**)

The nonparametric Kruskal–Wallis with Bonferroni’s post hoc test was applied in the quantitative analyses of the VD in different sectors. The statistical results are displayed in Tables 4 and Fig. 3. In the central ring, the VD of the control group, no-DR and mild-moderate NPDR groups were 28.38% (16.33, 37.78), 23.95% (18.44, 31.18) and 23.18% (15.77, 28.05), respectively, with no significant difference between the groups (*p* = 0.133). In the 3 mm radius, there were differences in the each quadrant (*p* < 0.05). Furthermore, the VD of the control group was bigger than the no-DR and mild-moderate NPDR groups (*p* < 0.005) in the superior and inferior quadrants, while no significant difference was found between the no-DR and mild-moderate NPDR groups (*p* > 0.005). In the temporal and nasal quadrants, the only difference was found between the control group and mild-moderate NPDR group (*p* = 0.002 and 0.006). In the 6 mm radius, there were differences in the superior, inferior and temporal quadrants (*p* = 0.031, 0.041, 0.028). Among them, the VD of the control group was bigger than the mild-moderate NPDR group (*p* = 0.044, 0.035, 0.035). However, the difference between the mild-moderate NPDR and control groups vs no-DR group was not significant (*p* = 0.60, 0.867, and 0.9111, respectively). In the wide-field scans (9 mm and 12 mm radius), there was no significant difference in the average VD between the groups in the ETDRS grid.

**Table 4:** Comparison of vascular density parameters between each group

	Control group	No-DR	Mild-Moderate DR	*P*	1 vs 2	1 vs 3	2 vs 3
Central ring	28.38(16.33,37.78)	23.95(18.44,31.18)	23.18(15.77.28.05)	0.133	0.251	0.082	1
3 mm radius							
Superior	70.47(64.89,74.86)	63.64(57.70,73.22)	62.73(59.43,68.72)	0.005	0.017	0.002	0.802
Inferior	69.73(62.89,73.53)	64.05(56.05,69.80)	61.23(54.36,67.89)	0.001	0.016	0.001	0.644
Temporal	66.86(62.54,72.67)	62.32(56.25,71.61)	61.32(49.56,67.59)	0.008	0.085	0.002	0.085
Nasal	66.68(62.43,70.51)	61.94(53.18,68.79)	59.71(53.89,65.47)	0.007	0.074	0.006	0.775
6 mm radius							
Superior	73.69(68.06,76.92)	67.66(59.09,72.66)	69.04(64.37,73.43)	0.031	0.068	0.044	0.824
Inferior	70.97(68.74,77.22)	67.81(61.66,76.75)	68.75(57.24,73.80)	0.041	0.288	0.035	0.812
Temporal	70.65(66.80,74.72)	67.50(62.20,74.72)	66.66(57.59,71.53)	0.028	0.429	0.035	0.134
Nasal	75.51(70.56,78.68)	72.90(67.74,80.96)	72.92(68.48,78.29)	0.699	0.533	0.372	0.907
9 mm radius							
Superior	70.45(64.44,73.14)	67.83(59.82,74.03)	66.79(59.82,74.03)	0.339	0.397	0.107	0.581
Inferior	68.40(62.12,75.07)	66.65(61.94,74.58)	67.24(60.01,70.15)	0.234	0.510	0.086	0.267
Temporal	65.79(57.18,68.76)	62.18(58.14,67.95)	60.96(53.62,64.88)	0.072	0.488	0.260	0.097
Nasal	76.55(70.15,80.59)	77.01(68.99,82.63)	75.86(70.18,81.46)	0.987	0.872	0.909	0.956
12 mm radius							
Superior	61.65(56.92,66.54)	62.11(54.44,69.58)	59.42(55.55,64.88)	0.388	0.966	0.234	0.231
Inferior	51.96(45.93,59.77)	51.13(42.71,57.50)	52.93(48.38,58.34)	0.430	0.378	0.834	0.222
Temporal	51.27(39.65,59.48)	48.91(40.93,56.74)	45.69(40.01,55.89)	0.839	0.911	0.649	0.585
Nasal	62.33(57.19,67.22)	61.27(53.60,70.16)	63.96(58.22,68.68)	0.689	0.790	0.558	0.416
0-12 mm radius	63.78(60.97,66.56)	60.92(57.98,67.10)	61.60(58.60,64.75)	0.145	0.731	0.041	0.589

### Correlation analysis

**Fig. 4:**
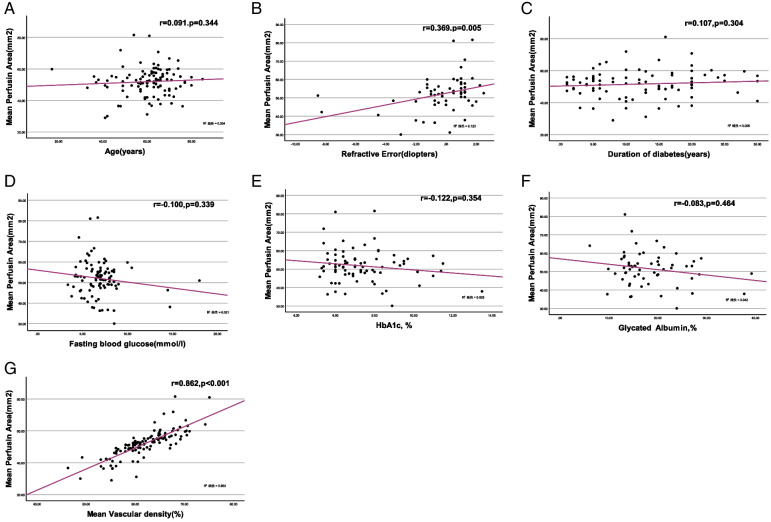
Scatterplots for simple linear regression between the mean perfusion area and age (**A**), refractive power (**B**), duration of diabetes (**C**), fasting blood glucose (**D**), HbA1c (**E**), glycaed albumin (**F**), and mean VD (**G**)

Among the diabetic patients, scatter plots of mean perfusion area and age, refractive power, duration of diabetes, fasting blood glucose, HbA1c, glycated albumin, and mean VD were drawn, followed by Spearman’s correlation analysis (Fig. 4). The results showed that the correlation between the mean PA and the refractive power and blood VD was statistically different (*r* = 0.369, *p* = 0.005 and *r* = 0.862, *p* < 0.001). Furthermore, regression analysis showed that the effect of VD on PA was statistically different (b = 1.311, t = 9.048, *p* < 0.001). On the other hand, the correlation between blood VD and age, refractive power, diabetes course, fasting blood glucose, glycosylated hemoglobin, and serum albumin were not statistically significant (all *p* > 0.05).

## Discussion

In this study, we used ultra-wide-angle SS-OCTA to analyze the changes in early retinal blood flow in patients with DR; data were compared to diabetic patients without DR and normal patients. The key findings indicated that wide-field OCTA might be useful in detecting early blood flow changes in the wide-angle area of the retina in diabetic patients.

Moreover, in the horizontal direction, the PA of the nasal area was larger than the temporal area of the same ring (except for 3 mm). Silva et al. [21] had evaluated the differences in peripheral vascular density using ultra-wide field (UWF) FA and found significantly lower perfusion density (PD) in the temporal half of the retina. This decrease in PD was attributed to a reduction in the number of vessels and a decrease in vessel diameter. Thus, the nasal region, which has a greater number of vessels and a thicker vessel diameter, will also has a higher PA than the corresponding temporal region. The morphology of the retinal capillary network was also linked to the metabolic demands of the neuroretina. Oxygen and metabolites need to be delivered efficiently. Therefore, the macula includes a high density of optic rods and ganglion cells and was considered to be an important region thereby displaying the highest vascular density and perfusion [22, 23]. The perfusion region of the upper area was observed to be greater than the area under the same ring in the vertical direction (3 mm and 12 mm). The difference in the vertical direction was different from the traditional 7-field ETDRS study because we divided the ETDRS area with the macular fovea as the center. However, during ultra-wide-angle SS-OCTA scanning, the upper and lower areas are susceptible to the patient's eyelids and eyelashes, which may cause partial coverage artifacts that affect the perfusion density and result in false-positive hypoperfusion [24]. Therefore, it is important to consider the eyelashes artifacts to quantify the peripheral PA, when using Wide-field SS-OCTA.

Comparison of the PA of mild-moderate NPDR, diabetes without DR and control groups showed that only the 3S, 3i, and 3 N regions had statistically significant differences. Previous studies have also found that as the incidence of DR increases, the retina VD in the central macular region decreases [25–27]. Yet, with progression, the macular arch will be damaged (enlargement of FAZ), blood VD will be reduced, and certain focal changes and even microangioma will occur [7, 28]. A meta-analysis found that the NDR group had expanded areas and perimeters of the FAZ relative to the healthy control group, as well as decreased perfusion density (PD) in both the superficial and deep capillary plexus of the macula (except parafoveal PD of the inner retina and foveal PD) and reduced radial peripapillary capillary PD [13].

Recent studies have also reported the association of capillary vessel density with DR and found that CVD changes may be a useful indicator of DR severity [29, 30]. However, the cumulative peripapillary perfusion is largely influenced by large vessels. Furthermore, since greater venular and arterial calibers are linked to DR development, the connection between capillary dropout and the DR stage can be muddled [31]. Retinal perfusion can also be improved in the early stages of diabetes [32]. Because of their broad diameters, venules can be especially effective confounders in evaluating DR in OCTA images [14]. Their findings also indicate that removing large vessels from perfusion density quantification improves NPDR diagnostic accuracy by using wide-field OCTA. In addition, the differences could not be apparent due to the small sampling size and the fact that only patients in the early stages of DR were included. Therefore, it may be possible to explain the early changes in retinal blood flow in the diabetic retina by eliminating large blood vessel detection methods or more accurate measurement methods and larger samples of data.

The retinal PA was shown to be strongly associated with blood VD, but there was no association with blood glucose indicators in this analysis. Previous research has found a connection between retinal blood and blood glucose levels [33]. In healthy subjects, a hyperglycemic/euinsulinemic clamp leads to an increase in ocular blood flow parameters [34]. Insulin administration decreases retinal blood flow by lowering blood glucose levels [35]. Also, blood flow is reduced toward normal during euglycemic conditions [36]. The majority of our diabetic patients visit the endocrinology department for blood glucose control to maintain a certain degree of stability. Besides, the non-significant variations or poor associations may be due to the ultra-wide-angle SS-OCTA that we used (12*12 mm), which scans a larger region and may include more retinal blood vessels.

In a recent study, the nonperfusion area in the peripheral sector showed the highest efficacy for determining the severity of DR [9]. Furthermore, wide-field SS-OCTA can depict changes in the microvascular perfusion status in DR, predicting the efficacy of anti-VEGF injection or planning a follow-up schedule.

There are a few limitations in the present study. First, this is a cross-sectional retrospective analysis. A prospective analysis should be conducted to better understand the association between parafoveal lesions' microvascular modifications and disease development in early-stage DR. Second, the sample size was insufficient to draw definitive conclusions. Third, the isolation of large vessels from capillary perfusion density calculation should be used to improved the difference. Finally, OCTA image artifacts will make it difficult to determine the state of the retinal microvasculature accurately.

In conclusion, wide-field OCTA imaging is useful for evaluating peripheral capillary perfusion in eyes with DR. Our data suggests that nasal quadrant PA is usually greater than the temporal quadrant PA at the same distance, and the PA in the 3 mm radius decreases in diabetic patients. However, the difference in the peripheral region, which may be affected by large blood vessels, is not very obvious. Therefore, more specific studies are required to further verify reported findings.

## Data Availability

The datasets used and/or analysed during the current study available from the corresponding author on reasonable request.

## Abbreviations

DR: Diabetic retinopathy
FAZ: Foveal avascular zone
CFP: Color fundus photography
FA: Fluorescein angiography
OCTA: Optical coherence tomography angiography
SS-OCT: Swept-source OCT
NPDR: Non-proliferative DR
PDR: Proliferative DR
PA: Perfusion area
VD: Blood vessel density
IRMA: Intraretinal microvascular abnormality
NVE: New arteries elsewhere
PD: Perfusion density
ETDRS: Early Treatment Diabetic Retinopathy Study
